# An assessment of the construct validity of the Child Health Utility 9D-CHN instrument in school-aged children: evidence from a Chinese trial

**DOI:** 10.1186/s12955-021-01840-7

**Published:** 2021-08-26

**Authors:** Mandana Zanganeh, Peymane Adab, Bai Li, Emma Frew

**Affiliations:** 1 Centre for Health Economics, Warwick Medical School, University of Warwick, Coventry, CV4 7AL UK; 2 Institute of Applied Health Research, College of Medical and Dental Sciences, University of Birmingham, Edgbaston, Birmingham, B15 2TT UK; 3 Centre for Exercise, Nutrition and Health Sciences, School for Policy Studies, University of Bristol, Bristol, UK

**Keywords:** Construct validity, CHU-9D, PedsQL, School-aged children, China

## Abstract

**Background:**

Although there is emerging data regarding the psychometric properties of the Child Health Utility-9D instrument, more evidence is required with respect to its validity for use in different country settings. The aim of this study was to examine the construct validity of the CHU-9D-CHN instrument in Chinese children.

**Methods:**

Baseline Health-Related Quality of Life (HRQoL) and demographic data were collected from children recruited to the CHIRPY DRAGON obesity prevention intervention randomised controlled trial in China. HRQoL was measured using the Chinese version of the CHU-9D instrument (CHU-9D-CHN) and the PedsQL instrument. CHU-9D-CHN utility scores were generated using two scoring algorithms [UK and Chinese tariffs]. Discriminant validity, known-group validity and convergent validity were evaluated using non-parametric test for trend, Kruskal–Wallis test and Spearman correlation coefficient analysis respectively.

**Results:**

Data was available for 1,539 children (mean age 6 years). The CHU-9D-CHN was sensitive to known group differences determined by the median PedsQL total score. Furthermore, the mean CHU-9D-CHN utility values decreased linearly with increasing levels of severity on each dimension of the PedsQL for emotional and social functioning domains. They decreased monotonically with increasing levels of severity on each dimension of the PedsQL for physical and school functioning domains (*p* < 0.001). Contrary to studies conducted in Western countries, and although not statistically significant, we found an indication that HRQoL, using both the CHU-9D-CHN and the PedsQL, was higher in children whose parents had lower levels of education, compared to those whose parents were university educated. The correlation between the CHU-9D-CHN utility values using UK and Chinese tariffs, and PedsQL total scores showed a statistically significant moderate positive correlation (Spearman’s rho = 0.5221, *p* < 0.001 and Spearman’s rho = 0.5316, *p* < 0.001), respectively. However, each CHU-9D-CHN dimension was either weakly, or very weakly correlated with each of the predetermined PedsQL domain functioning scores.

**Conclusions:**

Overall, the findings provide some support for the construct validity of the CHU-9D-CHN within a Chinese population aged 6–7 years. However, some uncertainty remains. We recommend future studies continue to test the validity of the CHU-9D in different country settings.

*Trial registration*: ISRCTN Identifier ISRCTN11867516, Registered on 19/08/2015

**Supplementary Information:**

The online version contains supplementary material available at 10.1186/s12955-021-01840-7.

## Background

Obesity prevention interventions have increasingly targeted primary school-aged children [1]. This has implications for the methods of outcome measurement within economic evaluation of these interventions as few instruments exist which are designed to generate utilities, for the construction of Quality-Adjusted Life Years (QALYs), in this age group [1]. Assessment of health status in children is unlike adults and requires a different conceptual approach. This is because of rapid rates of development in children, dependency on parents/caregivers and differences in disease epidemiology [2]. The assessment of each individual’s health related quality of life (HRQoL) relies on their subjective evaluation of functioning in different domains. It has been suggested that children’s subjective health reports are not reliable and are therefore of limited use [3]. However, research demonstrates that primary school-age children aged 8–10 years [4], and perhaps even younger [5], can adequately reflect and report their health state provided the instruments use appropriate language and the constructs are relevant to the age group. HRQoL instruments may either be self-administered or interviewer-administered by parents, caregivers or researchers. As the cognitive and language skills of young children are not completely developed, it is necessary to use interviewers to help with reading out the questions for the assessment of HRQoL in this age group.

Ideally, utility-based health-related quality of life in children should be measured using an instrument specifically designed for them [6]. Although there is no gold standard for measuring utility-based HRQoL in primary school-aged children, previous research has shown that the Child Health Utility-9D (CHU-9D) is an appropriate choice [7]. It is a preference-based instrument that generates utility values anchored between the values of 0 (being dead) and 1 (perfect health), with negative values denoting states worse than being dead. It is a generic instrument, not specific to any one condition or disease, and designed for application in economic evaluation of prevention, treatment and service programmes targeted at young people where the QALY is the desired outcome measure [8]. Although it has been used in populations with a wide age range (from 6 to 17 years) [9, 10], it was originally developed and validated for children aged 7–11 years in the UK [11, 12]. More recently its construct validity was demonstrated in 11–17 year olds in Australia [13] and Denmark [14].

The Paediatric Quality of Life Inventory TM (PedsQL) is a widely used HRQoL instrument validated for use with young children over 5 years old in diverse populations [15, 16]. It has good reliability and validity in both paediatric patients and healthy populations [15, 16]. The PedsQL is currently a non-preference based instrument which does not apply any explicit weighting between item domains and therefore cannot be used to generate utility values for the construction of QALYs. However, it would be expected to produce HRQoL values which move in the same direction as the utility values.

A UK study in children aged 5–6 years [9], an Australian study in children aged 11–17 years [13], and a Danish study in high-school students [14], found evidence of lower HRQoL in children from a lower socio-economic background. These studies, including a study from China found that there was a strong or moderate positive correlation between the CHU-9D utility values and PedsQL total scores [9, 13, 14, 17]. Although there is emerging evidence regarding the psychometric properties of the CHU-9D instrument [9, 13, 14], there is a dearth of instruments available for assessing HRQoL among Chinese children and more evidence is required on the CHU-9D before widespread use in China and in other settings with a large number of Chinese migrants such as Malaysia and Singapore. This is important because the measure may have different construct validity in different populations which might affect the results of health economic evaluations.

The aim of this study was therefore to assess the construct validity of the CHU-9D-CHN instrument in 6–7 year- old children in a Chinese setting, with the objectives being:

- To assess the known-group validity, referring to the principle that the CHU-9D-CHN should be able to demonstrate different scores for groups of children who are known to vary on HRQoL (e.g. socio-economic status [9, 13, 14]).
- To determine the discriminant or divergent validity of the instrument by exploring how the different dimensions of HRQoL that are theoretically not supposed to be related are actually related.
- To determine the convergent validity of the instrument, referring to the degree to which the CHU-9D-CHN and PedsQL capture a common construct of HRQoL [18].

## Methods

### Trial design and participants

The analysis presented uses data from the CHIRPY DRAGON cluster-randomised controlled trial assessing effectiveness and cost-effectiveness of a childhood obesity prevention intervention in Guangzhou, China [19, 20]. Children took part in baseline measurements in 2015 when they were 6–7 years old, and were followed up for 12 months. At baseline, a range of measurements were undertaken, including HRQoL measured using the PedsQL and CHU-9D-CHN; height; weight; gender; age (in months); and socio-economic factors. This study used the complete baseline data for 1,539 children to assess the CHU-9D-CHN in relation to the PedsQL.

All year-one students from non-boarding, state-funded (residents) primary schools/clusters (n = 353) located in the largest Southern Chinese city, Guangzhou were eligible for inclusion. The majority of Chinese children attend this type of school [21, 22]. A few private schools, mainly for children of foreign residents [21, 22], were not eligible. The trial study team randomly selected 40 schools using a random number generator and obtained permission to recruit from each school’s principal. Informed consent was then sought for each child participant from their parents/guardians. The sample size (1640 children) was based on being able to detect a difference of 0.17 units in the mean BMI z scores between arms in a cluster of 40 schools, with 80% power and at a 5% significance level.

All outcomes were collected at the individual level by independent and trained assessors (research staff) using standardised procedures and instruments. Data on participants’ date of birth and gender were obtained from school records.

### Anthropometric measurements

Height and weight measurements were undertaken without shoes and in light clothing. Standing height was measured at least twice with a TGZ-type height tester (Dalian). Weight was measured with an electronic scale (JH-1993 T, weighing Apparatus Co. Ltd., Dalian, China). Body mass index (BMI) was calculated as weight in kilograms divided by the square of height in metres (kg/m2). The WHO 2007 Growth charts were used to calculate BMI z-scores and to categorise the children into underweight, healthy weight, overweight and obese groups [23].

### Measurement of HRQoL

The Chinese version of the CHU-9D (CHU9D-CHN) [24] and PedsQL, which are both generic instruments, were chosen for the measurement of HRQoL. Both instruments were researcher-administered considering the young age of the participants.

The CHU-9D-CHN instrument combines nine dimensions of HRQoL: worried; sad; pain; tired; annoyed; schoolwork/homework; sleep; daily routine; and ability to join in activities [11, 25] (Additional file 1: Appendix 1). Each dimension comprises five severity levels, resulting in 1,953,125 unique health states associated with the measure. Individual responses from the questionnaires were transformed into utility weights derived from a UK general population sample using an algorithm developed by Stevens et al. [11, 25]. This presents a possible utility value set of between 0.33 (worst health state) and 1 (best health state). The CHU-9D-CHN instrument has a Chinese tariff set available for estimating utility values, but according to the instrument developers [personal communication], at the time of this study, the Chinese-specific preference weights were still in development and required further validation therefore it was recommended to use the UK tariff set, and to use the Chinese-tariff set as an exploratory analysis [26]. The Chinese-tariff set that was used was obtained using utility weights derived from a Chinese student population (mean age 13 years) presenting a possible utility value set of between − 0.09 (worst health state) and 1 (best health state) [26].

The PedsQL is a 23-item instrument comprising four domains: physical (8 items), emotional (5 items), social (5 items), and school (5 items) functioning [15]. Each item has five response options: never; hardly ever; sometimes; often; almost always. Emerging from the instrument is a score (transformed on to a 0–100 scale) for each domain and a score for total HRQoL. Decreasing value of the score indicates poorer HRQoL. For this study the validated Chinese version of the PedsQL 4.0 instrument was used [27]. The mean score for each of the four domains was calculated by summing the values for the relevant items and dividing by the number of items answered. This process generated a mean for the total score (mean of all items), for the physical health score (mean of physical functioning items) and for the psychosocial health score (mean of emotional, social and school functioning items).

### Known-group validity

The factors associated with HRQoL were explored. The relationship between HRQoL and weight status category (defined as either ‘overweight/obese vs. healthy/underweight’ or ‘underweight vs. healthy weight, overweight and obese’); and with gender were examined. HRQoL was assessed in relation to socio-economic status (SES) using the parent’s education level coded as a binary variable (did; did not obtain a university degree) and a categorical variable (school education; college vocational education; university undergraduate education; university postgraduate education). Mother/father’s education level was collected through a parent completed questionnaire at baseline and was the pre-specified proxy measure of SES in the primary analysis. Mother/father’s employment status was used as an alternative measure of SES as part of a sensitivity analysis. This was coded as a binary variable (did; did not work) and a categorical variable (working full time; working part time; unemployed or looking for work; looking after the family/house; other). Differences in HRQoL scores between groups were assessed using either the Kruskal–Wallis test (across all levels of categorical variables), or the non-parametric test for trend (across ordered categories of a variable). Non-parametric tests were used because the HRQoL variables did not follow a normal distribution (based on Kolmogorov–Smirnov test).

Statistical tests of difference were used to determine if the CHU-9D-CHN instrument was sensitive to identifying different scores between groups with known differences. The hypothesis was that studies from UK, Australian, and Danish settings reported a poorer HRQoL for children from lower socio-economic backgrounds [9, 13, 14], therefore we used SES for this analysis. Furthermore, the sample was split according to the median PedsQL total score. The mean (SD) CHU-9D-CHN utility values (using the UK and Chinese tariffs) were compared for children who had a score either on/above, or below, this median PedsQL score, using the t-test.

### Discriminant validity

To assess the discriminant validity, we examined how well the mean CHU-9D-CHN utility values corresponded with the options for each of the PedsQL dimensions, and for this, the mean CHU-9D-CHN utility value was estimated for each level of PedsQL response on every dimension. The hypothesis was that the mean CHU-9D-CHN utility values would decrease linearly or monotonically with increasing severity on each of the PedsQL dimensions.

### Convergent validity

Convergent validity was explored, using statistical tests of association, to determine how the CHU-9D-CHN correlated with the PedsQL measure. Graphical means (scatter plots), along with fitted regression line and 95% CIs, for the CHU-9D-CHN utility values and the PedsQL total scores were used to show the relationship between the instruments. Then, using the Spearman’s rho statistic, the correlation coefficient between the CHU-9D-CHN utility values and the PedsQL total scores was calculated. The hypothesis was that there would be a strong or moderate positive correlation between the CHU-9D-CHN utility values and PedsQL total scores [9, 13, 14].

Spearman’s Rank correlation coefficient Rs is a technique which can be used to summarise the strength and direction (negative or positive) of a relationship between two instruments. The result is always between 1 and − 1. The meaning of the strength of the correlation using the guide for the value of Rs [28] is: 0.00–0.19: a very weak correlation; 0.20–0.39: a weak correlation; 0.40–0.69: a moderate correlation; 0.70–0.89: a strong correlation; 0.90–1.00: a very strong correlation.

The content and coverage of the two instruments were further examined by assessing the correlation between individual CHU-9D-CHN dimensions and the PedsQL domains that were conceptually similar, as follows:

- Physical functioning: pain, tired, sleep, daily routine
- Emotional functioning: worried, sad, annoyed
- Social functioning: ability to join in activities
- School functioning: school work/home work

All statistical analyses were undertaken in 2019, using Stata version 13.

### Ethics

The study was funded through a philanthropic donation from Zhejiang Yong Ning Pharmaceutical Ltd Company from 2014 to 2019. Full ethics approvals were obtained from the Life and Health Sciences Ethical Review Committee at the University of Birmingham (2nd March, 2015) and the Ethical Committee of Guangzhou Centre for Disease Control and Prevention (1st December, 2014). The CHIRPY DRAGON trial was registered on 19th of August, 2015 (registration number: ISRCTN11867516).

## Results

### Participant characteristics

Complete data (including PedsQL total score and its sub-scales; CHU-9D-CHN dimensions and utility value; height and weight (converted to BMI z-score and weight status); gender; age; and parents’ education level) were available for 1539 out of 1640 children (93.8% of those who consented and participated in study measurements) and are described in Table 1.

**Table 1:** Characteristics of the study population

Characteristics	
Gender: n (%)	
Male	831 (54.0)
Female	708 (46.0)
Age (years): mean (SD)	6.6 (0.42)
Measures of socio-economic status	
Maternal university education: n (%)	
Yes	963 (62.6)
No	576 (37.4)
Maternal education level: n (%)	
1 School education	296 (19.2)
2 Occupation college	280 (18.2)
3 University undergraduate education	847 (55.1)
4 University postgraduate education	116 (7.5)
Paternal university education: n (%)	
Yes	1005 (65.3)
No	534 (34.7)
Paternal education level: n (%)	
1 School education	247 (16.2)
2 Occupation college	287 (18.6)
3 University undergraduate education	824 (53.5)
4 University postgraduate education	181 (11.7)
Weight status: n (%)	
Underweight	75 (4.9)
Healthy weight	1189 (77.2)
Overweight	165 (10.7)
Obese	110 (7.2)
Underweight/Healthy weight compared to Overweight/Obese: n (%)	
Underweight/Healthy weight	1264 (82.1)
Overweight/Obese	275 (17.9)
BMI: mean (SD)	15.45 (2.13)
BMI z-score: mean (SD)	− 0.12 (1.29)
CHU-9D-CHN mean utility value (SD)	
CHU-9D: using UK tariff	0.937 (0.068)
CHU-9D: using Chinese tariff	0.920 (0.094)
PedsQL mean score (SD)	
PedsQL Total scale score	82.92 (11.21)
PedsQL Physical functioning	83.67 (13.15)
PedsQL Emotional functioning	81.69 (17.54)
PedsQL Social functioning	84.09 (15.30)
PedsQL School functioning	81.77 (15.36)

*BMI* body mass index, *SD* standard deviation

**Fig. 1:**
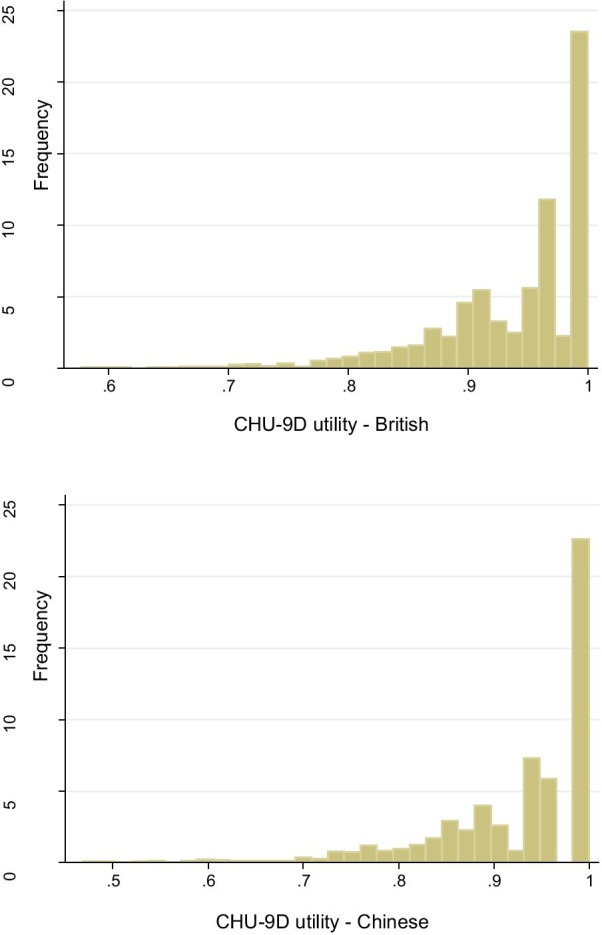
Distribution of the CHU-9D utility scores based on both British and Chinese tariffs

The mean age of the children was 6.6 years (SD = 0.42) and 54% were male. Around a third of parents were educated to below university degree. The mean BMI z-score was -0.12 (SD = 1.29), whilst more than 17% of the children were either overweight (10.7%) or living with obesity (7.2%); comparable to national data from China for overweight and obesity in the same age group (20.4%) [21]. The mean utility scores of the total sample was, on average, slightly higher for CHU-9D-CHN using the UK tariff (mean = 0.937 [SD = 0.068]) compared to using the Chinese tariff (mean = 0.920 [SD = 0.094]) (Fig. 1). The mean total PedsQL score was 82.92 (SD = 11.21). Data on parental employment status was available for 1,539 children and is presented in Additional file 1: Appendix 2.

### Known-group validity

Table 2 summarises the CHU-9D-CHN utility values and PedsQL total scores according to the weight status, gender of the children, and SES of the children’s parents. The direction of the relationships was similar between instruments. Of interest, the mean utility scores using both UK and Chinese tariffs and mean PedsQL total scores were all marginally higher for children who were overweight/obese compared to those who were not. These differences were not statistically significant. The CHU-9D-CHN reported a slightly higher mean utility score for girls compared to boys (*p* = 0.003 and *p* = 0.004 respectively) consistent with the mean PedsQL total score which was also higher in girls, although this difference was not statistically significant. Both HRQoL instruments reported scores that were marginally higher in children whose parents did not have a university education (lower SES) compared to those who did but again, these differences were not statistically significant. The analyses were re-run using parental employment status as an alternative proxy for SES and the results were similar (Additional file 1: Appendix 3).

**Table 2:** Comparison of mean (SD) and median (IQR) for CHU-9D-CHN and PedsQL scores according to respondent characteristics

	Number (%)	CHU-9D utility, UK tariff	CHU-9D utility, Chinese tariff	PedsQL total score
		Mean (SD), median (IQR)	Mean (SD), median (IQR)	Mean (SD), Median (IQR)
Gender				
Male	831 (54.0)	0.932 (0.072), 0.952 (0.897–1.000)	0.914 (0.098), 0.939 (0.873–1.000)	82.29 (11.72), 83.69 (75.00–91.30)
Female	708 (46.0)	0.943 (0.063), 0.963 (0.909–1.000)	0.927 (0.089), 0.955 (0.881–1.000)	83.66 (10.54), 85.86 (77.17–91.30)
*p*-value*		0.003*	0.004*	0.06
Mother’s university education				
Yes	963 (62.6)	0.936 (0.068), 0.956 (0.903–1.000)	0.920 (0.091), 0.943 (0.876–1.000)	82.58 (11.29), 83.69 (76.08–91.30)
No	576 (37.4)	0.938 (0.068), 0.963 (0.903–1.000)	0.921 (0.099), 0.952 (0.874–1.000)	83.49 (11.07), 85.86 (77.17–91.30)
*p*-value*		0.27	0.42	0.08
Mother education level				
1 School education	296 (19.2)	0.937 (0.070), 0.963 (0.895–1.000)	0.921 (0.096), 0.953 (0.870–1.000)	83.06 (11.18), 85.86 (76.08–91.30)
2 Occupation college	280 (18.2)	0.940 (0.067), 0.963 (0.907–1.000)	0.919 (0.102), 0.945 (0.879–1.000)	83.95 (10.96), 85.86 (78.26–91.30)
3 University undergraduate education	847 (55.1)	0.937 (0.068), 0.958 (0.903–1.000)	0.920 (0.091), 0.943 (0.876–1.000)	82.58 (11.37), 83.69 (76.08–91.30)
4 University postgraduate education	116 (7.5)	0.932 (0.070), 0.952 (0.901–1.000)	0.919 (0.092), 0.942 (0.885–1.000)	82.59 (10.71), 84.23 (75.00–89.13)
*p*-value**		0.27	0.36	0.19
Father’s university education				
Yes	1005 (65.3)	0.936 (0.068), 0.955 (0.902–1.000)	0.920 (0.091), 0.943 (0.876–1.000)	82.90 (11.06), 84.78 (76.08–91.30)
No	534 (34.7)	0.939 (0.069), 0.963 (0.904–1.000)	0.921 (0.100), 0.955 (0.876–1.000)	82.97 (11.51), 85.86 (76.08–91.30)
*p*-value*		0.17	0.38	0.61
Father education level				
1 School education	247 (16.2)	0.931 (0.075), 0.963 (0.892–1.000)	0.911 (0.110), 0.943 (0.864–1.000)	82.27 (11.65), 83.69 (75.00–91.30)
2 Occupation college	287 (18.6)	0.946 (0.062), 0.963 (0.915–1.000)	0.928 (0.090), 0.955 (0.882–1.000)	83.57 (11.36), 85.86 (76.08–92.39)
3 University undergraduate education	824 (53.5)	0.937 (0.067), 0.960 (0.903–1.000)	0.921 (0.090), 0.943 (0.877–1.000)	83.11 (11.14), 84.78 (76.08–91.30)
4 University postgraduate education	181 (11.7)	0.932 (0.072), 0.952 (0.897–1.000)	0.916 (0.096), 0.943 (0.870–1.000)	81.91 (10.65), 83.69 (76.08–89.13)
*p*-value**		0.42	0.63	0.53
Weight status groups				
Underweight	75 (4.9)	0.942 (0.067), 0.963 (0.908–1.000)	0.923 (0.092), 0.938 (0.873–1.000)	82.47 (12.06), 85.86 (72.82–92.39)
Healthy weight	1189 (77.2)	0.936 (0.069), 0.962 (0.900–1.000)	0.919 (0.095), 0.943 (0.876–1.000)	82.84 (11.13), 83.69 (76.08–91.30)
Overweight	165 (10.7)	0.941 (0.064), 0.963 (0.909–1.000)	0.925 (0.086), 0.955 (0.874–1.000)	83.18 (11.65), 85.86 (76.08–91.30)
Obese	110 (7.2)	0.939 (0.071), 0.962 (0.914–1.000)	0.921 (0.096), 0.943 (0.890–1.000)	83.69 (10.94), 86.95 (77.17–91.30)
*p*-value**		0.73	0.89	0.29
Weight status groups				
Underweight/healthy weight	1264 (82.1)	0.936 (0.069), 0.963 (0.901–1.000)	0.919 (0.095), 0.943 (0.875–1.000)	82.82 (11.18), 83.69 (76.08–91.30)
Overweight/Obese	275 (17.9)	0.940 (0.067), 0.964 (0.909–1.000)	0.923 (0.090), 0.944 (0.876–1.000)	83.38 (11.35), 85.86 (76.08–91.30)
*p*-value**		0.38	0.66	0.27

*IQR* inter-quartile range, *SD* standard deviation

*Kruskal–Wallis test

**Non-parametric test for trend

The mean (SD) utility scores for children who had a PedsQL score that was less than or equal to the median value, compared to those with PedsQL scores greater than or equal to the median value were 0.909 (0.075) and 0.967 (0.043) respectively for the UK tariff; and 0.881 (0.106) and 0.961 (0.056) respectively for the Chinese tariff (*p* < 0.001).

### Discriminant validity

Table 3 summarises the mean CHU-9D-CHN utility values across the dimension levels of the PedsQL. The majority of children reported themselves in good health, with the largest proportion reporting themselves at the highest level for all dimensions of the PedsQL. In general, the mean CHU-9D-CHN utility values corresponded well, decreasing linearly with increasing levels of severity on each dimension of the PedsQL for emotional and social functioning domains, and decreasing monotonically with increasing levels of severity on each dimension of the PedsQL for physical and school functioning domains (*p* < 0.001). This result was statistically significant (*p* < 0.001) for each of the dimensions.

**Table 3:** Mean CHU-9D-CHN utility value by each level of PedsQL dimension

PedsQL dimensions	Level	n (%)	Mean (SD) CHU9D utility	*p*-value*
*Physical functioning*				
Walking trouble	Never	963 (62.5)	0.948 (0.063)	< 0.001
	Hardly ever	148 (9.6)	0.927 (0.072)	
	Sometimes	273 (17.8)	0.920 (0.074)	
	Often	80 (5.2)	0.927 (0.063)	
	Almost always	75 (4.9)	0.902 (0.083)	
Running trouble	Never	1101 (71.5)	0.947 (0.061)	< 0.001
	Hardly ever	189 (12.3)	0.918 (0.071)	
	Sometimes	184 (11.9)	9.912 (0.083)	
	Often	49 (3.1)	0.909 (0.088)	
	Almost always	16 (1.2)	0.887 (0.076)	
Exercise trouble	Never	1078 (70.1)	0.948 (0.061)	< 0.001
	Hardly ever	171(11.1)	0.919 (0.077)	
	Sometimes	217 (14.1)	0.918 (0.072)	
	Often	40 (2.6)	0.904 (0.095)	
	Almost always	33 (2.1)	0.879 (0.087)	
Carrying trouble	Never	674 (43.8)	0.950 (0.060)	< 0.001
	Hardly ever	183 (11.9)	0.920 (0.079)	
	Sometimes	437 (28.4)	0.932 (0.069)	
	Often	129 (8.4)	0.928 (0.075)	
	Almost always	116 (7.5)	0.921 (0.073)	
Showering trouble	Never	1280 (83.2)	0.943 (0.064)	< 0.001
	Hardly ever	106 (6.8)	0.913 (0.073)	
	Sometimes	72 (4.7)	0.908 (0.081)	
	Often	38 (2.5)	0.935 (0.071)	
	Almost always	43 (2.8)	0.870 (0.096)	
Housework trouble	Never	1087 (70.7)	0.948 (0.061)	< 0.001
	Hardly ever	157 (10.3)	0.913 (0.078)	
	Sometimes	189 (12.2)	0.917 (0.074)	
	Often	48 (3.1)	0.922 (0.071)	
	Almost always	58 (3.7)	0.883 (0.093)	
Feeling pain	Never	1056 (68.7)	0.951 (0.058)	< 0.001
	Hardly ever	149 (9.7)	0.909 (0.080)	
	Sometimes	287 (18.6)	0.912 (0.074)	
	Often	36 (2.3)	0.899 (0.092)	
	Almost always	11 (0.7)	0.837 (0.134)	
Feeling tired	Never	1044 (67.8)	0.949 (0.059)	< 0.001
	Hardly ever	135 (8.8)	0.923 (0.068)	
	Sometimes	292 (18.9)	0.917 (0.070)	
	Often	46 (3.1)	0.873 (0.106)	
	Almost always	22 (1.4)	0.842 (0.122)	
*Emotional functioning*				
Feeling fearful	Never	965 (62.7)	0.949 (0.059)	< 0.001
	Hardly ever	142 (9.2)	0.928 (0.071)	
	Sometimes	303 (19.7)	0.921 (0.071)	
	Often	82 (5.3)	0.910 (0.085)	
	Almost always	47 (3.1)	0.871 (0.105)	
Feeling sad	Never	1114 (72.4)	0.949 (0.058)	< 0.001
	Hardly ever	128 (8.3)	0.916 (0.069)	
	Sometimes	247 (16.1)	0.908 (0.078)	
	Often	37 (2.4)	0.886 (0.103)	
	Almost always	13 (0.8)	0.824 (0.143)	
Feeling angry	Never	923 (59.9)	0.953 (0.054)	< 0.001
	Hardly ever	154 (10.1)	0.925 (0.074)	
	Sometimes	338 (22.0)	0.921 (0.070)	
	Often	92 (6.0)	0.885 (0.095)	
	Almost always	32 (2.0)	0.850 (0.111)	
Feeling insomnia	Never	997 (64.7)	0.951 (0.056)	< 0.001
	Hardly ever	118 (7.6)	0.926 (0.074)	
	Sometimes	243 (15.9)	0.913 (0.078)	
	Often	120 (7.8)	0.905 (0.084)	
	Almost always	61 (4.0)	0.883 (0.087)	
Feeling worried	Never	949 (61.7)	0.951 (0.060)	< 0.001
	Hardly ever	134 (8.7)	0.932 (0.067)	
	Sometimes	330 (21.4)	0.917 (0.074)	
	Often	72 (4.7)	0.899 (0.080)	
	Almost always	54 (3.5)	0.886 (0.086)	
*Social functioning*				
Difficulties socialising	Never	1138 (73.9)	0.946 (0.061)	< 0.001
	Hardly ever	149 (9.7)	0.917 (0.079)	
	Sometimes	189 (12.3)	0.913 (0.079)	
	Often	45 (2.9)	0.906 (0.075)	
	Almost always	18 (1.2)	0.860 (0.105)	
Other children did not want to socialise	Never	927 (60.3)	0.949 (0.060)	< 0.001
	Hardly ever	199 (12.9)	0.926 (0.072)	
	Sometimes	311 (20.2)	0.918 (0.074)	
	Often	71 (4.6)	0.916 (0.075)	
	Almost always	31 (2.0)	0.885 (0.096)	
Other children mocked	Never	1104 (71.7)	0.947 (0.060)	< 0.001
	Hardly ever	180 (11.7)	0.919 (0.074)	
	Sometimes	195 (12.7)	0.913 (0.078)	
	Often	39 (2.5)	0.883 (0.101)	
	Almost always	21 (1.4)	0.882 (0.083)	
Inability to socialise	Never	947 (61.6)	0.949 (0.058)	< 0.001
	Hardly ever	189 (12.4)	0.924 (0.076)	
	Sometimes	325 (21.1)	0.920 (0.074)	
	Often	49 (3.1)	0.890 (0.094)	
	Almost always	29 (1.8)	0.887 (0.089)	
Difficulties for tracking	Never	961 (62.5)	0.949 (0.060)	< 0.001
	Hardly ever	155 (10.1)	0.928 (0.065)	
	Sometimes	327 (21.2)	0.919 (0.074)	
	Often	66 (4.3)	0.911 (0.084)	
	Almost always	30 (1.9)	0.867 (0.117)	
*School functioning*				
Difficulties concentrating	Never	921 (59.9)	0.951 (0.057)	< 0.001
	Hardly ever	142 (9.3)	0.919 (0.068)	
	Sometimes	347 (22.5)	0.918 (0.077)	
	Often	85 (5.5)	0.917 (0.073)	
	Almost always	44 (2.8)	0.883 (0.113)	
Difficulties memorising	Never	761 (49.4)	0.952 (0.061)	< 0.001
	Hardly ever	194 (12.7)	0.923 (0.081)	
	Sometimes	423 (27.5)	0.928 (0.065)	
	Often	120 (7.8)	0.916 (0.067)	
	Almost always	41 (2.6)	0.883 (0.084)	
Difficulties studying/catching	Never	959 (62.4)	0.951 (0.058)	< 0.001
	Hardly ever	174 (11.3)	0.919 (0.068)	
	Sometimes	302 (19.6)	0.918 (0.074)	
	Often	64 (4.1)	0.906 (0.081)	
	Almost always	40 (2.6)	0.869 (0.104)	
Absent from school due to sickness	Never	1062 (69.1)	0.942 (0.065)	< 0.001
	Hardly ever	139 (9.0)	0.927 (0.071)	
	Sometimes	298 (19.4)	0.932 (0.068)	
	Often	36 (2.3)	0.884 (0.114)	
	Almost always	4 (0.2)	0.892 (0.083)	
Absent from school due to hospitalisation	Never	1079 (70.2)	0.942 (0.065)	< 0.001
	Hardly ever	169 (10.9)	0.930 (0.074)	
	Sometimes	265 (17.3)	0.925 (0.070)	
	Often	16 (1.0)	0.902 (0.094)	
	Almost always	10 (0.6)	0.872 (0.110)	

*Non-parametric test for trend

*SD* standard deviation

### Convergent validity

**Fig. 2:**
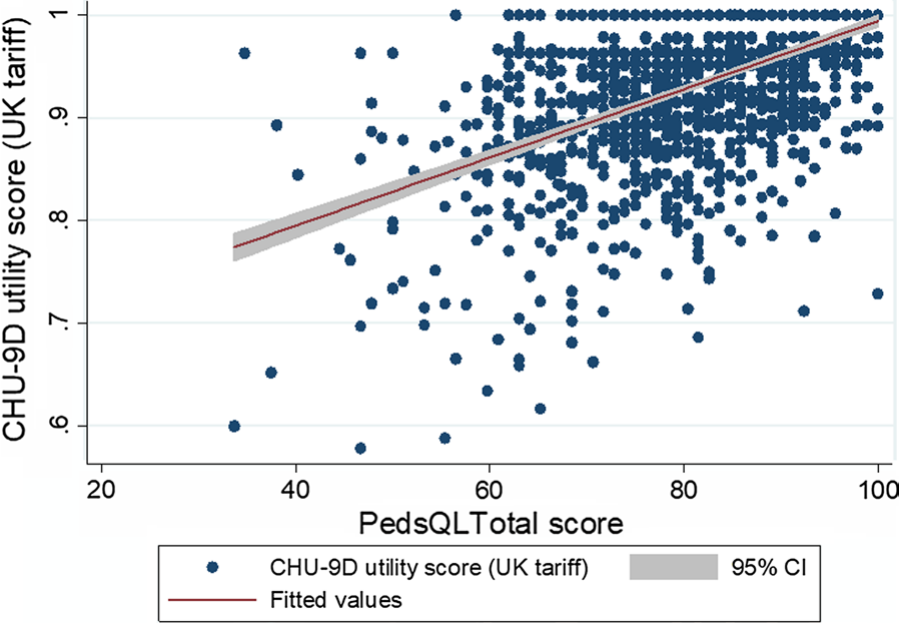
Relationship between CHU-9D utility scores (UK tariff) and PedsQL total scores

**Fig. 3:**
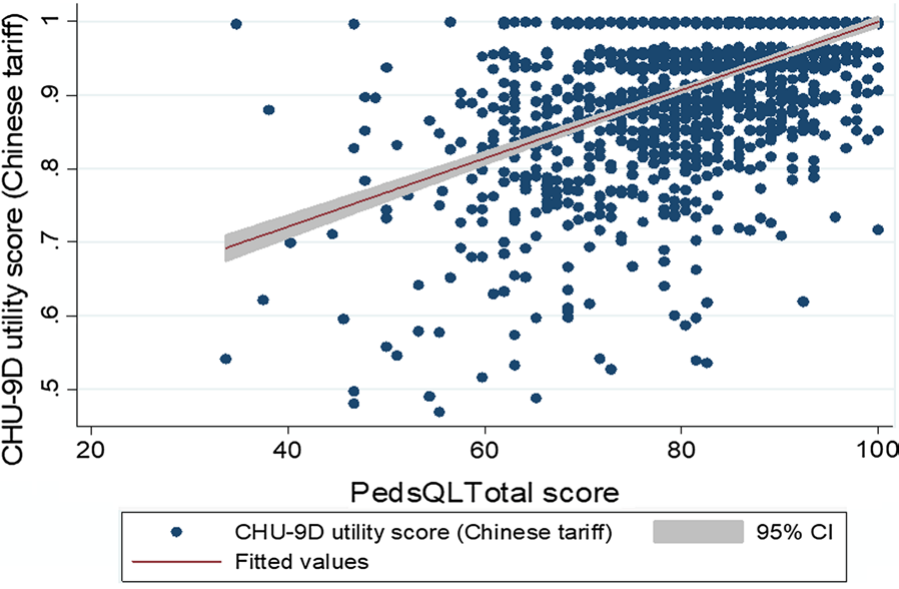
Relationship between CHU-9D utility scores (Chinese tariff) and PedsQL total scores

Figure 2 shows a scatter plot comparison of the relationship between the CHU-9D-CHN utility values (using UK tariff) and the PedsQL total scores. Some anomalies were apparent. For instance, one child reported a high CHU-9D-CHN utility score of 0.963, yet had a low PedsQL total score of 34.78. However, in general, there was a moderate association between the instruments with higher CHU-9D-CHN utility values corresponding with higher PedsQL total scores and the CHU-9D-CHN utility values and PedsQL total scores converging towards the highest end of the scale. Figure 3 shows a scatter plot comparison of the relationship between the CHU-9D-CHN utility values (using Chinese tariff) and the PedsQL total scores. Figure 2 is similar to Fig. 1 but some wider anomalies were apparent. For instance, one child reported a high CHU-9D-CHN utility score of 0.996, yet had a low PedsQL total score of 34.78, and another child reported a low CHU-9D utility score of 0.535, yet had a high PedsQL total score of 82.60. However, in general, again there was a moderate association between the instruments with higher CHU-9D-CHN utility values corresponding with higher PedsQL total scores and the CHU-9D-CHN utility values and PedsQL total scores converging towards the highest end of the scale. Overall, the correlation between the CHU-9D-CHN utility values and PedsQL total scores showed a statistically significant moderate positive correlation for the UK tariff set (Spearman’s rho = 0.5221, *p* < 0.001) and the Chinese tariff set (Spearman’s rho = 0.5316, *p* < 0.001).

The content and coverage of the two instruments were further compared by examining the correlation between each of the CHU-9D-CHN dimensions and the theoretically similar PedsQL domain functioning scores (Table 4). Using conventional cut-off values for Spearman’s rho, each CHU-9D-CHN dimension was either weakly, or very weakly correlated with each of the predetermined PedsQL domain functioning scores. Since the CHU-9D-CHN dimensions were labelled with 1 as highest level and 5 as lowest level, the signs on the coefficients were consistently negative. All correlations were significant at the 0.01 level.

**Table 4:** Correlation between CHU-9D-CHN dimensions and PedsQL domain functioning scores

	Worried	Sad	Pain	Tired	Annoyed	Schoolwork	Sleep	Daily routine	Activities	CHU-9D utility (British tariff)	CHU-9D utility (Chinese tariff)
Physical functioning	− 0.174	− 0.116	− 0.203	− 0.273	− 0.137	− 0.275	− 0.149	− 0.196	− 0.250	0.425	0.415
Psychosocial functioning	− 0.286	− 0.254	− 0.218	− 0.309	− 0.257	− 0.335	− 0.239	− 0.217	− 0.234	0.513	0.529
Emotional functioning	− 0.260	− 0.239	− 0.191	− 0.277	− 0.220	− 0.251	− 0.288	− 0.196	− 0.152	0.451	0.476
Social functioning	− 0.195	− 0.213	− 0.169	− 0.238	− 0.193	− 0.247	− 0.132	− 0.143	− 0.215	0.383	0.385
School functioning	− 0.201	− 0.127	− 0.140	− 0.194	− 0.175	− 0.276	− 0.117	− 0.159	− 0.177	0.342	0.350
PedsQL total score	− 0.277	− 0.229	− 0.239	− 0.334	− 0.241	− 0.353	− 0.233	− 0.236	− 0.270	0.522	0.531

Absolute values reported. All Spearman’s correlations are statistically significant (all *p* value < 0.001)

The paired comparison of the CHU-9D-CHN utility scores, using the UK and Chinese tariffs illustrated that the mean UK utility values (0.937, SD 0.068) were on average, marginally higher than the Chinese utility values (0.920, SD 0.094) and this difference was statistically significant (*p* < 0.001) (Fig. 1).

## Discussion

### Statement of principal findings

With respect to known-group validity, contrary to studies conducted in Western countries [9, 13, 14], and although not statistically significant, we found an indication that HRQoL, using both the CHU-9D-CHN and the PedsQL, was higher in children whose parents had lower levels of education, compared to those whose parents were university educated. The CHU-9D-CHN demonstrated different scores according to the median PedsQL total score. For the discriminant validity, the mean CHU-9D-CHN utility values decreased linearly with increasing levels of severity on each dimension of the PedsQL for emotional and social functioning domains. They decreased monotonically with increasing levels of severity on each dimension of the PedsQL for physical and school functioning domains (*p* < 0.001).With respect to convergent validity, although there was a moderate significant positive correlation between CHU-9D-CHN utility values and PedsQL total scores, the correlation between individual CHU-9D-CHN dimensions and the theoretically similar PedsQL domains were weak or very weak. We also found the mean utility to be higher using the UK tariff-set in comparison to the Chinese tariff-set and this finding was expected given the underlying differences in valuation methodology and corresponding scale values.

### Strengths and limitations of this study

Strengths include the large sample size (1539 children), diverse population (selected to include a range of socio-economic backgrounds) and standardised data collection procedures as part of the randomised controlled trial. Furthermore, this study was one of the very few studies worldwide and the first study in China that collected utility-based HRQoL information in children as young as 6 years. It used both UK and Chinese tariffs for calculating the utility scores and reports on the psychometric properties of the CHU-9D-CHN in direct comparison to the widely used PedsQL instrument.

The study had some limitations. Data analysis was limited to data collected as part of the trial therefore the analysis was limited to an assessment of the CHU-9D-CHN validity in relation to the socio-demographic and economic variables collected within the trial and the PedsQL. However, there is no ‘gold standard’ instrument to assess construct validity in this context, and the PedsQL is a widely used HRQoL instrument validated for use with young children in diverse populations [15, 16]. Although the CHU-9D has only been validated in children and adolescents from 7 to 18 years old, we have experience of using this in large studies with children as young as 6-years old [7, 29]. Furthermore, as the only preference based HRQOL instrument that has been designed exclusively with children for children, it was the most appropriate instrument to measure utility-based HRQOL at the time. Within the study, the CHU-9D was interviewer-administered because of the wide range of reading skills within the study population. This may have influenced the child responses, but we minimised this by using trained data collectors to interview participants individually in a private and familiar environment, away from other children and school staff. The interviewers were given age-appropriate communication skills training and read out the questions verbatim, providing clarification only when a child had language difficulties. Since the study was conducted, a new proxy version of the CHU-9D has been developed that is designed to be completed on behalf of children aged 5–7 years by an appropriate caregiver. Further research will determine if CHU-9D proxy-values are a more appropriate method for assessing HRQoL in this age group, instead of interviewer-administered CHU-9D self-assessed values. The evidence on whether proxy-reported values should be used for children is mixed but there does seem to be a consensus that where possible, self-report should be used, and this is especially the case for when a judgement is required on un-observable signs or symptoms [30]. In terms of further limitations, as there are cultural, infrastructural and other system-related differences between China and other countries, the generalisability of results to other contexts, particularly to developed country settings, could be questionable.

### Comparison with other studies

Regarding the discriminant validity, some findings were in line with a previous study reported from a UK setting [9]. With respect to known-group validation, an interesting result was that, unlike a UK study in children aged 5–6 years [9], an Australian study in children aged 11–17 years [13], and a Danish study in high-school students [14], this study found no evidence of lower HRQoL in children from a lower socio-economic background—in fact the direction of effect was the reverse. This might be because the measures of SES are not equivalent in China and other countries. As a country in economic transition, educational level and employment may not reflect the same status as we see in the West. Also, as a communist country, SES measures may have less significance and no association with quality of life. The results of this study also differed from another study in a Chinese setting that reported a statistically significant trend for higher HRQoL scores (using PedsQL) in children who had parents with higher levels of education [21]. Two main differences were noted: in this study, all children were 6–7 years old (compared to 5–12 years old in the other study) and were from state schools, compared to the other study where 30% attended private schools for children of economic migrants. It is also worth noting that the study was conducted within a large urban city in China, where educational levels are generally higher, and a large proportion of parents reported being University educated.

For the convergent validity, the findings were similar to the previous studies in the UK and China [9, 17]. The weak, or very weak correlation between the individual dimensions of each instrument might be because these individual dimensions describe something that is quite specific and different while appearing superficially similar. Also, perhaps there are overlaps between elements in some domains/dimensions which are resulting in the weak correlations, whilst the overall scores are better correlated.

## Conclusions

Overall, the findings provide some support for the construct validity of the CHU-9D-CHN within a Chinese population aged 6–7 years. This is because (1) the CHU-9D-CHN was sensitive to known differences determined by the PedsQL median score; (2) the mean CHU-9D-CHN utility values decreased linearly with increasing levels of severity on each dimension of the PedsQL for emotional and social functioning domains, and they decreased monotonically with increasing levels of severity on each dimension of the PedsQL for physical and school functioning domains (*p* < 0.001); and (3) there was a moderate significant positive correlation between CHU-9D-CHN utility values and PedsQL total scores. However, there still remains areas of uncertainty as the CHU-9D-CHN dimensions were only weakly correlated with theoretically similar PedsQL dimensions and it is unclear why this was the case.

Overall we recommend future studies continue to test the validity of the CHU-9D in China and in other countries sharing similar cultures or SES- profiles to China. This is important because the measure may have different construct validity in different populations which might affect the results of health economic evaluations.

## Supplementary Information

**Additional file 1.** Supplementary material.

## Data Availability

The dataset and materials used in this study are available from the corresponding author upon request.

## Abbreviations

BMI: Body mass index
CHU-9D: Child health utility 9 dimension
CHU-9D-CHN: Child Health Utility 9 dimension Chinese version
HRQoL: Health-related quality of life
PedsQL™: Pediatric quality of life inventory™
QALY: Quality-adjusted life years
SD: Standard deviation
SES: Socio-economic status
UK: United Kingdom
WHO: World Health Organisation
