# Supplementary material for: An assessment of the construct validity of the Child Health Utility 9D-CHN instrument in school-aged children: evidence from a Chinese trial
Source: Health Qual Life Outcomes. 2021 Aug 26;19:205. doi: 10.1186/s12955-021-01840-7 (PMC8394170; doi:10.1186/s12955-021-01840-7)
Supplement: Supplementary file 1 — Additional file 1. Supplementary material. [file 12955_2021_1840_MOESM1_ESM.docx]

**Appendices**

**Appendix 1: CHU-9D**

Thinking about today….

| **A1. Worried** |
| --- |
| O I don’t feel worried today |
| O I feel a little bit worried today |
| O I feel a bit worried today |
| O I feel quite worried today |
| O I feel very worried today |
| **A2. Sad** |
| O I don’t feel sad today |
| O I feel a little bit sad today |
| O I feel a bit sad today |
| O I feel quite sad today |
| O I feel very sad today |
| **A3. Pain** |
| O I don’t have any pain today |
| O I have a little bit of pain today |
| O I have a bit of pain today |
| O I have quite a lot of pain today |
| O I have a lot of pain today |
| **A4. Tired** |
| O I don’t feel tired today |
| O I feel a little bit tired today |
| O I feel a bit tired today |
| O I feel quite tired today |
| O I feel very tired today |
| **A5. Annoyed** |
| O I don’t feel annoyed today |
| O I feel a little bit annoyed today |
| O I feel a bit annoyed today |
| O I feel quite annoyed today |
| O I feel very annoyed today |
| **A6. Schoolwork/Homework (Such as reading, writing, doing lessons)** |
| O I have no problems with my schoolwork/homework today |
| O I have a few problems with my schoolwork/homework today |
| O I have some problems with my schoolwork/homework today |
| O I have many problems with my schoolwork/homework today |
| O I can’t do my schoolwork/homework today |
| **A7. Sleep** |
| O Last night I had no problems sleeping |
| O Last night I had a few problems sleeping |
| O Last night I had some problems sleeping |
| O Last night I had many problems sleeping |
| O Last night I couldn’t sleep at all |
| **A8. Daily routine (Things like eating, having a bath./shower, getting dressed)** |
| O I have no problems with my daily routine today |
| O I have a few problems with my daily routine today |
| O I have some problems with my daily routine today |
| O I have many problems with my daily routine today |
| O I can’t do my daily routine today |
| **A9. Able to join in activities (Things like playing out with your friends, doing sports, joining in things)** |
| O I can join in with any activities today |
| O I can join in with most activities today |
| O I can join in with some activities today |
| O I can join in with a few activities today |
| O I can join in with no activities today |

**Appendix 2 Characteristics of the study population**

| **Characteristics** | |
| --- | --- |
| **Measures of socio-economic status** | |
| **Maternal work: n (%)** | |
| Yes  No | 1190 (77.3)  349 (22.7) |
| **Maternal employment status: n (%)** | |
| 5 Working full-time  4 Working part time  3 Unemployed or looking for work  2 Looking after the family/house  1 Other | 1043 (67.8)  147 (9.5)  15 (1.0)  280 (18.2)  54 (3.5) |
| **Paternal work: n (%)** | |
| Yes  No | 1421 (92.3)  118 (7.7) |
| **Paternal employment status: n (%)** | |
| 5 Working full-time  4 Working part time  3 Unemployed or looking for work  2 Looking after the family/house  1 Other | 1378 (89.5)  43 (2.8)  10 (0.7)  17 (1.1)  91 (5.9) |

**Appendix 3 Comparison of mean (SD) and median (IQR) for CHU-9D-CHN and PedsQL scores according to respondent characteristics**

|  | **Number**  **(%)** | **CHU-9D Utility, UK tariff**  **Mean (SD), Median (IQR)** | **CHU-9D Utility, Chinese tariff**  **Mean (SD), Median (IQR)** | **PedsQL total score**  **Mean (SD), Median (IQR)** |
| --- | --- | --- | --- | --- |
| **Mother’s employment** | | | | |
| Yes  No | 1190 (77.3)  349  (22.7) | 0.936 (0.069), 0.963 (0.900-1.000)  0.940 (0.065), 0.965 (0.909-1.000) | 0.919 (0.095), 0.943 (0.876-1.000)  0.923 (0.091), 0.946 (0.878-1.000) | 82.51 (11.49), 84.09 (75.00-91.30)  83.04 (11.13), 84.78 (76.08-91.30) |
| **p-value*** |  | 0.60 | 0.73 | 0.50 |
| **Mother employment status** | | | | |
| 5 Working full-time  4 Working part time  3 Unemployed or looking for work  2 Looking after the family/house  1 Other | 1043 (67.8)  147  (9.5)  15  (1.0)  280  (18.2)  54  (3.5) | 0.936 (0.069), 0.962 (0.900-1.000)  0.936 (0.073), 0.963 (0.893-1.000)  0.892 (0.104), 0.915 (0.812-0.963)  0.942 (0.063), 0.963 (0.914-1.000)  0.940 (0.058), 0.952 (0.904-1.000) | 0.919 (0.094), 0.943 (0.875-1.000)  0.919 (0.107), 0.953 (0.881-1.000)  0.872 (0.122), 0.892 (0.760-0.996)  0.925 (0.090), 0.949 (0.881-1.000)  0.925 (0.083), 0.939 (0.880-1.000) | 82.99 (10.94), 84.78 (76.08-91.30)  81.74 (11.93), 82.60 (75.00-91.30)  81.15 (12.16), 84.78 (71.73-89.13)  82.73 (11.40), 84.78 (76.08-91.30)  83.46 (12.43), 86.95 (76.08-92.39) |
| **p-value**** |  | 0.57 | 0.65 | 0.60 |
| **Father’s employment** | | | | |
| Yes  No | 1421 (92.3)  118 (7.7) | 0.937 (0.068), 0.963 (0.899-1.000)  0.938 (0.065), 0.964 (0.903-1.000) | 0.920 (0.094), 0.943 (0.876-1.000)  0.922 (0.095), 0.950 (0.880-1.000) | 82.79 (11.23), 84.78 (76.08- 91.30)  84.48 (10.92), 86.95 (76.08-92.39) |
| **p-value*** |  | 0.93 | 0.91 | 0.07 |
| **Father employment status** | | | | |
| 5 Working full-time  4 Working part time  3 Unemployed or looking for work  2 Looking after the family/house  1 Other | 1378 (89.5)  43  (2.8)  10  (0.7)  17  (1.1)  91  (5.9) | 0.937 (0.068), 0.963 (0.903-1.000)  0.923 (0.087), 0.952 (0.893-0.978)  0.948 (0.040), 0.957 (0.928-0.963)  0.931 (0.063), 0.940 (0.897-0.978)  0.937 (0.068), 0 .963 (0.897-1.000) | 0.921 (0.093), 0.943 (0.876-1.000)  0.895 (0.119), 0.937 (0.848-0.958)  0.936 (0.054), 0.946 (0.896-0.996)  0.898 (0.112), 0.916 (0.851-0.996)  0.924 (0.095), 0.955 (0.880-1.000) | 82.81 (11.25), 84.78 (76.08-91.30)  82.30 (10.65), 82.60 (76.08-91.30)  87.06 (8.20), 90.21 (83.69-91.30)  85.80 (10.91), 90.21 (75.00-94.56)  83.95 (11.21), 85.86 (77.17-92.39) |
| **p-value**** |  | 0.97 | 0.84 | 0.17 |

*Kruskal-Wallis test; **non-parametric test for trend
